# *Actinomyces* lesions and acute inflammation predominate in osteonecrosis of the jaw associated with osteoclast-suppressing therapy in contrast to non-medication-related osteonecrosis

**DOI:** 10.1007/s10096-026-05501-9

**Published:** 2026-04-06

**Authors:** Marjo Kivelä-Rajamäki, Hanna Välimaa, Jussi Furuholm, Caj Haglund, Timo Sorsa, Jaana Hagström, Asko Järvinen

**Affiliations:** 1 Department of Infectious Diseases, Inflammation Centre, Faculty of Medicine, Helsinki University Hospital and University of Helsinki, Helsinki, Finland; 2 Meilahti Vaccine Research Centre MeVac, Department of Infectious Diseases, Helsinki University Hospital and University of Helsinki, Helsinki, Finland; 3 Department of Oral and Maxillofacial Diseases, Faculty of Medicine, University of Helsinki, Helsinki University Hospital, Helsinki, Finland; 4 Department of Surgery and Translational Cancer Medicine Research Program, University of Helsinki and Helsinki University Hospital, Helsinki , Finland; 5 Department of Oral Medicine, Karolinska Institutet, Stockholm, Sweden; 6 Department of Pathology, Helsinki University Hospital, Helsinki, Finland; 7 Institute of Dentistry/Oral Pathology, Faculty of Medicine, University of Turku, Turku, Finland

**Keywords:** medication-related osteonecrosis of the jaw, MRONJ, *Actinomyces*, MMP-8, inflammation, osteoclast-suppressive antiresorptive medication

## Abstract

**Purpose:**

Patients receiving antiresorptive therapy for cancer bone metastases/lesions or osteoporosis may develop osteonecrosis of the jaw. The pathogenesis remains unclear, but the condition has been associated with *Actinomyces*, co-pathogens, and pro-inflammatory activity. However, there are limited studies comparing these factors between medication-related osteonecrosis and non-medication-related osteonecrosis.

**Methods:**

This is a single-centre retrospective study of 98 patients with jawbone biopsies due to medication-related osteonecrosis treated with bisphosphonates and/or denosumab, and 93 patients with non-medication-related osteonecrosis during 2002–2020. We reviewed their medical records and analysed tissue samples for *Actinomyces* colonies, inflammation and proinflammatory collagenase matrix metalloproteinase-8 (MMP-8) using immunohistochemistry.

**Results:**

*Actinomyces* colonies were more prevalent in medication-related osteonecrosis (85%) compared to non-medication-related cases (51%; *p* < 0.001) and were mainly associated with necrotic bone. Inflammation was present in most samples; however, in medication-related osteonecrosis, it was predominantly acute (58%) and rarely chronic (5%), whereas in non-medication-related biopsies, inflammation was acute in 38% and chronic in 27% of biopsies (*p* < 0.001). Enhanced MMP-8 immunoreactivity was observed in 61% of medication-related osteonecrosis vs. 38% in non-medication-related biopsies (*p* = 0.002), and MMP-8 was especially expressed alongside actinomycotic lesions (57% vs. 34%; *p* < 0.001). In multivariable logistic regression, the medication-related group was independently associated with a higher prevalence of *Actinomyces* colonies.

**Conclusion:**

Medication-related osteonecrosis was independently linked to a higher prevalence of *Actinomyces* compared to non-medication-related osteonecrosis. Furthermore, acute inflammation and collagenase activity seemed to be effects of *Actinomyces*-associated infection. The presence of *Actinomyces* may suggest immunological differences between these osteonecrosis types.

**Supplementary Information:**

The online version contains supplementary material available at 10.1007/s10096-026-05501-9.

## Introduction

Patients with malignant bone metastases/lesions or osteoporosis receiving osteoclast-suppressive antiresorptive medications such as bisphosphonates (BP), denosumab, or possibly romosozumab, may develop medication-related osteonecrosis of the jaw (MRONJ) as a side effect [1]. This condition was first recognised over twenty years ago [2, 3], but its pathogenesis remains controversial. A recent study showed that osteonecrosis developed within five years in cancer patients, ranging from 1.4% to 6.6% depending on the medication [4]. The treatment of osteonecrosis is often empirical and, according to the American Association of Oral and Maxillofacial Surgeons (AAOMS) protocol, includes surgical debridement and antimicrobial therapy [5].

Oral pathogens [6–9], particularly *Actinomyces* species [8–14], and triggering events such as jaw procedures or trauma [1], have been associated with antiresorptive medication-related osteonecrosis. Bone immunity has also attracted attention. Macrophages are vital for initiating immune responses [15] and regulating bone formation [16]. Osteoclast-suppressive medication has been shown to increase the proinflammatory activity of osteal macrophages [17], supporting the development of osteonecrosis in a bacterial environment [17, 18] as osteocytes undergo necroptosis [19]. Inflammation-triggered tissue damage promotes bacterial invasion [18, 20]. Osteoclasts, which are bone remodelling and osteoimmune cells [21, 22] are suppressed by antiresorptive drugs as a primary pharmacological target through different modes of action. This suppression may impair bone homeostasis, thereby facilitating osteonecrosis [1, 5].

*Actinomyces* species are anaerobic/microaerophilic, Gram-positive, often branching rods that colonise tissue boundaries and when invading tissues, they cause actinomycotic infections [23, 24] with specific lesions (“sulphur granules”) [23, 24] which are frequently seen with multiple oral *Actinomyces* taxa [23]. They can be initial pathogens in biofilms [23] and participate in polymicrobial infections [23, 24].

MMP-8 is a proinflammatory collagenase. By cleaving collagen and other extracellular matrix substrates, as well as non-matrix bioactive mediators such as serpins, complement components, and insulin receptors, MMP-8 participates in immune-cascade processes and modifies them [25]. Elevated MMP-8 activity has been linked to the severity of infections [26–28].

We aimed to explore the relationship between *Actinomyces*, inflammation, and the immune cascade activity by analysing MMP-8 immunoreactivity, and to examine potential differences in inflammatory response between MRONJ and non-medication-related osteonecrosis (non-MRONJ) in jawbone tissue samples. Currently, there are no sufficient studies to confirm whether the incidence of invasive *Actinomyces* differs between these patient groups. Only a preliminary report involving just 11 patients with non-medication-related osteonecrosis suggested possible differences in the presence of *Actinomyces* compared to bisphosphonate-related osteonecrosis [14].

## Materials and methods

This retrospective clinical study involved patients from Helsinki University Hospital (serving a population of 1.8 million) who were diagnosed and biopsied for osteonecrosis of the jaw between 2002 and 2020.

### Ethical Considerations

The study was conducted in accordance with the Helsinki Declaration. Research permits to use human tissue samples in medical research were obtained from the Finnish Medicines Agency, FIMEA, and Helsinki Biobank. The study protocol was approved by the Helsinki University Hospital Ethical Committee of Medical Research, and the hospital´s research authorisation for clinical data collection and use in scientific research.

### Clinical data

This single-centre retrospective study examined patients with antiresorptive medication-related osteonecrosis of the jaw (MRONJ) treated with bisphosphonates (BP) and/or denosumab for cancer metastases or osteoporosis. The diagnosis was made according to AAOMS criteria [5]. Patients included had available clinical data and a tissue biopsy confirming osteonecrosis during debridement surgery. The study group consisted of patients with MRONJ linked to osteoclast-suppressive antiresorptive medication, while the control group (non-MRONJ) included patients with osteonecrosis without prior antiresorptive treatment. The MRONJ group was further divided into patients with cancer bone metastases/lesions and those with osteoporosis, whereas the non-MRONJ group was subdivided into patients with osteoradionecrosis or osteonecrosis caused by other factors. Clinical data and bacterial culture results were obtained from patient records.

Tissue samples.

Histological samples were initially obtained from patients to diagnose jawbone lesions. Of MRONJ biopsies, 25% were taken before 2014, when the American Association of Oral and Maxillofacial Surgeons (AAOMS) established the term dental status pretreatment protocol strategies for MRONJ [5]. Earlier position papers (2007 and 2009) had suggested preliminary management strategies for at-risk patients, and only 2% of the samples were taken before 2007 [5]. 41% of the MRONJ samples were collected in 2014–2016, and 34% in 2017–2020. In the non-MRONJ group, 48% of the samples were collected before 2014, 30% between 2014 and 2016, and 22% in 2017–2020.

Samples were fixed in formalin, decalcified with EDTA, embedded in paraffin tissue (FFPE) blocks, and stored according to the laboratory’s clinical protocol in the Helsinki Biobank for future scientific research, with patients’ written informed consent.

### Histology and immunohistochemistry

The Haematoxylin and Eosin (H&E) and PAS-stained slides were re-evaluated to confirm the diagnoses, including the presence of *Actinomyces* colonies and the inflammatory process. Immunohistochemical staining was performed following the manufacturer’s protocol for the Envision Flex kit (K8000), followed by rinsing, dehydrating, and coverslipping.

Antibodies (active MMP-8 [29] and tissue inhibitor of metalloproteinase 1 (TIMP-1)) for immunohistochemistry are described in the Supplementary Table 1.

The staining process was performed using an Autostainer 480 S from LabVision Corp., Fremont, CA, USA, or an Autostainer Link48 from Dako North America Inc., California, USA. After the staining procedure was completed, the specimen underwent dehydration (aqua-70%-96%-ethanol-xylene) and was then mounted (Pertex Histolab media, Askim, Sweden).

**Image 1:**
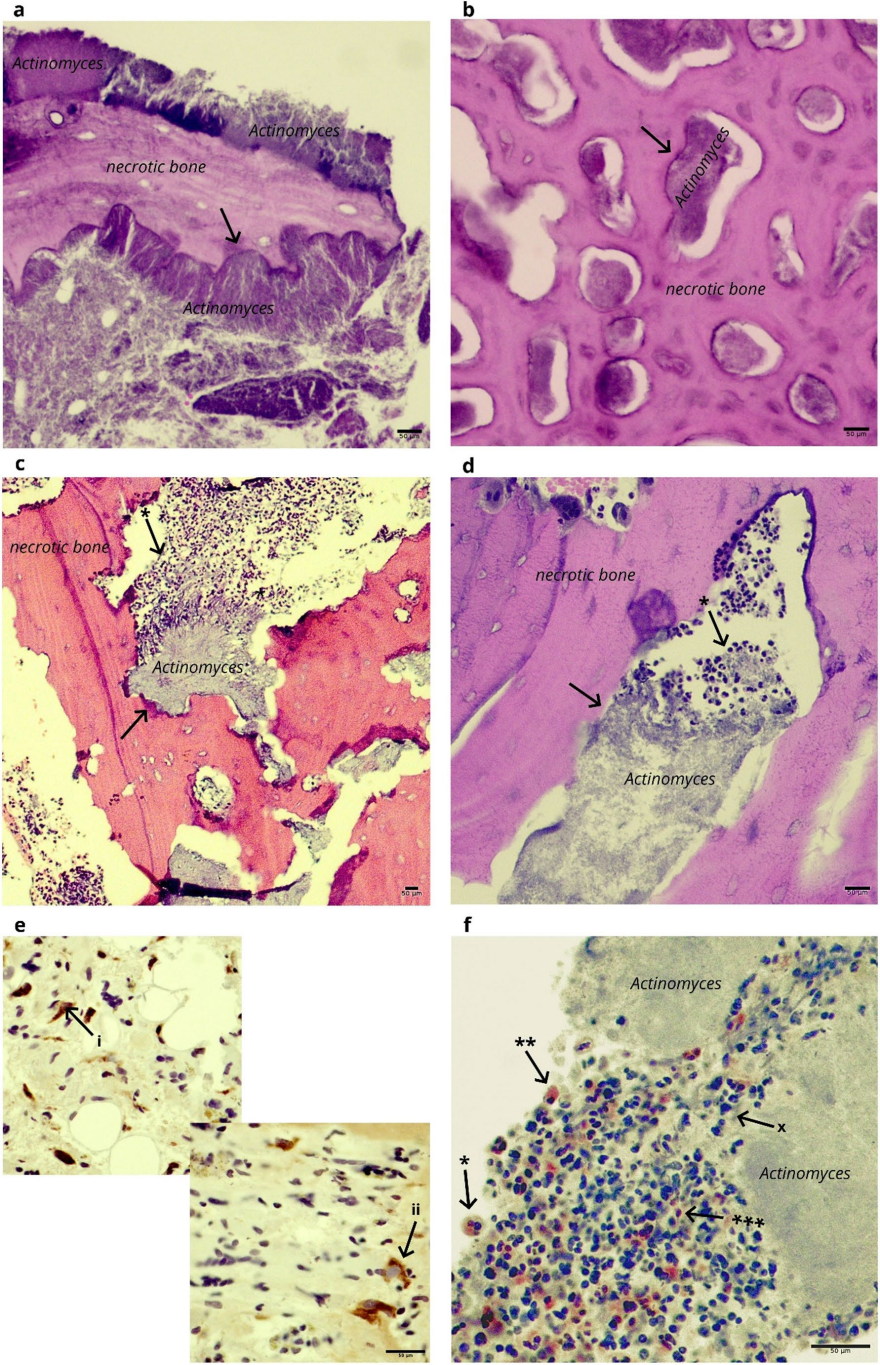
(**a**-**f**) Histological and Immunohistochemical Staining Panel:

The interpretation of histopathology and immunopositivity is illustrated in Image 1.

Inflammation was classified as acute (PMN leucocyte proliferation), chronic (non-PMN leucocyte proliferation, mainly lymphocyte and/or plasma cell proliferation), or mixed, including both.

*Actinomyces* colonies were assessed and semi-quantified through visual scoring as none, mild to moderate, or high levels, and their localisation was determined, focusing on their connection to the necrotic bone, whether lining, invading the bone, or showing no connection.

MMP-8 immunopositive cells were semi-quantified as low (none to marginal, < 10 positive cells) or elevated (≥ 10 positive cells). Results were further classified as: 0 = none; <10 positive cells = marginal; 10–19 positive cells = moderate; and ≥ 20 positive cells = high staining. Association of MMP-8 immunopositive cells with *Actinomyces* colonies was also measured, along with MMP-8 expression in PMN cells (yes/no) and in other cell types.

In immunohistochemistry, we counted all MMP-8-positive cells in the samples; the osteonecrosis samples were small and area-based methods, such as cells/mm^2^, were not suitable for counting.

Two of the samples were technically unsuccessful and could not be analysed for MMP-8. In nine patients, only original sectioned slides with H&E/PAS staining were available, without tissue blocks, making immunohistochemistry for MMP-8 unfeasible.

Two independent researchers evaluated the samples without knowledge of the clinical data. The background of the necrosis could not be visualised from the coded samples. An experienced oral pathologist on the research team guided the visual scoring and semi-quantification. In a few samples, when the researcher’s classification did not match the oral pathologist’s criteria, the oral pathologist made the final decision on the scoring.

Statistics.

SPSS Statistics software version 29 (IBM Corp., Chicago, IL, USA) was used for the statistical analysis. The presence of *Actinomyces* colonies, MMP-8 immunoexpression, and acute inflammation in the tissue sample were the primary outcome variables. The Pearson Chi-Square (2-sided) test or the Mann-Whitney U-test was used to assess statistical significance of differences between groups, with a significance level set at *p* < 0.05.

Statistical analyses were performed comparing the MRONJ and non-MRONJ groups, the MRONJ cancer and osteoporosis subgroups, BP versus denosumab +/- previously used BP groups, and non-MRONJ osteoradionecrosis versus other causes of osteonecrosis subgroups.

Multivariable binary logistic regression analyses were performed to examine the association between MRONJ (vs. non-MRONJ) and the primary outcomes, adjusting for potential confounders, including age, sex, smoking, corticosteroid use, and comorbidities. To address potential within-patient dependency, a sensitivity analysis including only the first biopsy per patient was also performed.

## Results

A total of 191 patients with histological tissue samples from jaw osteonecrosis met our inclusion criteria (Table 1): The medication-related osteonecrosis (MRONJ) group included 98 patients: 65 with cancer-related bone metastases/lesions (14 with breast cancer, 35 with prostate cancer, 9 with multiple myelomas, and 7 with other cancers) and 33 with osteoporosis. The non-MRONJ group included 93 patients: 21 with osteoradionecrosis and 72 with other causes of osteonecrosis, predominantly jaw infections (*n* = 66). One patient with osteopetrosis was excluded. Overall, 203 bone samples were analysed (Supplementary Table 2). After opening the clinical code linking biopsies and patient records, 12 samples were identified as second lesions from patients: 9 from MRONJ cancer patients, 2 from MRONJ osteoporosis patients, and one from a non-MRONJ osteoradionecrosis patient. All second samples were from different jaw areas, and 8 of the samples were from different years.

**Table 1:** Characteristics of patients with jaw osteonecrosis who underwent tissue biopsy (*N* = 191), divided into groups of antiresorptive medication-related osteonecrosis (*n* = 98) and non-antiresorptive medication-related osteonecrosis (*n* = 93), along with their subgroups

Osteonecrosis type:	MRONJ: *N* = 98	MRONJ Cancer *n* = 65	MRONJ Osteoporosis *n* = 33	Non-MRONJ: *N* = 93	Non-MRONJ Osteo- radionecrosis *n* = 21	Non-MRONJ Other osteonecrosis *n* = 72	*p*-value^a)^
**Age (years)**: *mean ± SD*	**70** ***±*** **12**	68 *±* 11	76 *±* 13	**55** ***±*** **17**	64 *±* 9	53 *±* 18	*< 0.001*
**Male (%)**	**55**	75	15 ^b)^***	**60**	62	60	*NS*
**Smoking: (%)**							
Smoker	**28**	27	29	**54**	58	54	*0.002*
Ex-smoker	**30**	34	16	**21**	16	21	*NS*
**Comorbidities: (%)**							
*Diabetes*	**14**	12	18	**12**	0	15	*NS*
*Cardiovascular*	**41**	31	61	**39**	29	42	*NS*
*Asthma/COPD*	**10**	11	9	**11**	5	13	*NS*
*Kidney disease*	**2**	2	3	**2**	0	3	*NS*
*Autoimmune diseases*	**31**	17	58	**11**	0	14	*0.001*
*Heavy alcohol consumption*	**7**	5	12	**14**	19	13	*NS*
*Neurological disease*	**13**	5	30	**10**	14	8	*NS*
*Psychiatric disease*	**2**	0	6	**5**	5	6	*NS*
*Artificial joint*	**11**	9	15	**2**	5	1	*0.019*
*IV drug abuse*	**1**	2	0	**2**	0	3	*NS*
*HIV*	**1**	0	3	**1**	0	1	*NS*
**Co-medication: (%)**							
*Cytotoxic therapy*	**39**	59	0	**5**	19	1	*< 0.001*
*Inhaled steroid*	**4**	2	9	**8**	10	7	*-*
*Any inhaled asthma/COPD therapy*	**13**	14	12	**11**	14	10	*NS*
*Systemic steroid*	**51**	60	33	**10**	14	8	*< 0.001*
*Ca/vitamin D*	**66**	66	66	**7**	19	3	*< 0.001*
*Hormone blocking*	**45**	66	3	**4**	10	3	*< 0.001*
*Immuno-modulators*	**27**	20	39	**8**	10	7	*0.004*
**Antiresorptive drug: (%)**							
*Bisphosphonate*	**54**	36	85	**-**	-	-	-
*Denosumab*	**17**	25	3	**-**	-	-	-
*Both*	**29h**	39	12	**-**	-	-	-

### Clinical findings

Most patients were male in both the MRONJ (55%) and non-MRONJ groups (60%). In the MRONJ-group, 75% of cancer patients were male, whereas 85% of osteoporosis patients were female (*p* < 0.001). MRONJ patients had a mean age of 70 years, which was significantly older than that of the non-MRONJ group (55 years, *p* = 0.001). Smoking was less common in the MRONJ group compared to the non-MRONJ group (*p* = 0.002). Smoking status could not be retrieved from 16 patients. Patients with MRONJ had rheumatoid arthritis or autoimmune diseases more frequently (*p* = 0.001), particularly among osteoporosis patients. Artificial joint prostheses were more frequent in the MRONJ-group (*p* = 0.019). (Table 1)

Among patients with MRONJ, 54% had received only bisphosphonates (BP), 17% only denosumab, and 29% both medications. Among osteoporosis patients, 85% had been treated solely with BP (Table 1), and they had received significantly longer cumulative antiresorptive treatment (*p* < 0.001) than cancer patients (Table 2). Only three osteoporosis patients had received high-dose BP, and none of them had received high-dose denosumab. Of cancer patients, 64% had received high-dose BP, and 61% high-dose denosumab. (Table 2)

**Table 2:** Cumulative duration of antiresorptive medication (months) among patients with medication-related osteonecrosis of the jaw (*N* = 97). Subgroups include patients with malignant disease (cancer) with bone metastases/lesions (*n* = 64) and those with osteoporosis (*n* = 33)

Antiresorptive therapy duration (months)	MRONJ mean ± SD	MRONJ Cancer mean ± SD	MRONJ Osteoporosis mean ± SD	*p*-value
**Total**	49 *± 42*	36 *± 27*	76 *± 54*	*< 0.001*
**High**,** IV** ***BP***	**25 ± 21** *n* = 44	**22 ± 18** *n* = 41	**59 ± 35** *n* = 3	*0.013*
**Low**,** oral** ***BP***	**55 ± 49** *n* = 48	**23 ± 25** *n* = 18	**74 ± 50** *n* = 30	*< 0.001*
**High dose** ***denosumab***	**24 ± 12** *n* = 39	**24 ± 12** *n* = 39	-	-
**Low dose** ***denosumab***	**26 ± 9** *n* = 6	**30** *n* = 1	**25 ± 10** *n* = 5	*NS*

High dose *denosumab* = 120 mg/ every 4 weeks subcutaneous, Low dose *denosumab* = 60 mg/every 6 months subcutaneous. **Statistics**: *p-value* is measured with *Independent-Samples Mann-Whitney U test* between cancer vs. osteoporosis groups. a) Individual patients may have received several different antiresorptive therapies. From one patient we could not count reliably the duration of the antiresorptive therapy

Abbreviations: *MRONJ*, medication-related osteonecrosis of the jaw; *BP*, bisphosphonates; *NS*, not significant.

Cytotoxic therapy, systemic corticosteroids, hormone-blocking agents, and calcium and vitamin D supplementation were significantly more common in the MRONJ group than in the non-MRONJ group (Table 1). Within the MRONJ group, 59% of cancer patients had received cytotoxic therapy, whereas none of the osteoporosis patients had. In the non-MRONJ subgroup with osteoradionecrosis, 19% had received prior cytotoxic therapy. Within the MRONJ subgroups, 60% of cancer patients, but none of the osteoporosis patients, had received high-dose corticosteroids. Other patients who had received corticosteroids had received low doses of 5–10 mg/day, equivalent to prednisolone, for at least 4 weeks (Table 1). In the non-MRONJ group, two patients with other causes of osteonecrosis had previously received high-dose corticosteroids.

### Clinical and diagnostic findings of osteonecrosis

Patients on antiresorptive medication predominantly exhibited severe or advanced osteonecrosis (Stages 2–3 [5]). Only 7% of MRONJ patients were classified as Stage 1, while 60% were Stage 2 and 30% Stage 3. Samples from the MRONJ group were collected, on average, between 20 and 47 months after the triggering event (Supplementary Table 2). Laboratory results showed no significant differences in C-reactive protein (CRP) or blood leucocyte levels between the MRONJ and non-MRONJ groups. However, patients with MRONJ had notably lower haemoglobin levels than those without (*p* < 0.001; Supplementary Table 3). Six patients with MRONJ and two patients with non-MRONJ had bacteraemia. A total of 25 patients did not undergo laboratory testing. Culture results from routine sampling taken during or before surgery were available for 147 patients, either from necrotic bone (*n* = 62), an abscess or fistula at the necrosis site (*n* = 57), or from the purulent area adjacent to the necrosis. *Streptococcus anginosus*-group was significantly more frequently identified in MRONJ cultures compared to non-MRONJ (*p* = 0.018). Other virulent oral pathogens showed no statistically significant differences between groups. *Staphylococcus aureus* was rarely found, and in 11/12 positive samples, it was only marginally positive in culture. *Actinomyces* species were isolated in just 26 specimens (Table 3).

**Table 3:** Bacterial culture results from patients with jaw osteonecrosis categorised into groups of antiresorptive medication-related osteonecrosis (*n* = 92) and non-medication-related osteonecrosis of the jaw (*n* = 55)

Bacterial culture	MRONJ:	Non-MRONJ	*p*-value^a)^
*Streptococcus anginosus (%)*	**45**	**29**	***0***,***018***
*Actinomyces sp. (%)*	**17**	**18**	*NS*
*Streptococcus viridans (%)*	**67**	**64**	*NS*
*Anaerobe/facult.anaerobe g-rod* *incl. Porphyromonas gingivalis (%)*	**74**	**67**	*NS*
*Parvimonas micra (%)*	**29**	**16**	*NS*
*Prevotella intermedia (%)*	**60**	**46**	*NS*
*Fusobacterium nucleatum (%)*	**37**	**29**	*NS*
*Staphylococcus aureus (low) (%)*	**3**	**9**	*NS*
*Staphylococcus coag. neg sp. (%)*	**3**	**15**	*(0*,*020)*
*Enterococcus faecalis (%)*	**5**	**7**	*NS*
*Lactobacillus sp. (%)*	**9**	**9**	*NS*
*Oral anaerobes (%)*	**41**	**44**	*NS*
*Oral aerobes (%)*	**55**	**64**	*NS*
*Candida sp. (%)*	**24**	**29**	*NS*

**Osteonecrosis type in lesions: MRONJ: subgroups cancer (with bone metastases/lesions) and osteoporosis; non-MRONJ osteonecrosis: subgroups osteoradionecrosis**,** other (causes of) osteonecrosis.** a) Statistics: *p-value* is measured with *Pearson Chi-square (2-sided sig)* between MRONJ- vs. non-MRONJ-groups

Abbreviations: *MRONJ*, medication-related osteonecrosis of the jaw; *NS*, not significant; facult., facultative; g-rod, gram negative rod; coag.neg, coagulase negative.

Computer tomography (CT) was available for 58% of patients in the MRONJ group (53% in the cancer group and 69% in the osteoporosis group), and for 39% in the non-MRONJ group (41% in the osteoradionecrosis group and 39% in other causes of osteonecrosis). In 58% of patients in the MRONJ group, osteonecrosis was diagnosed radiologically using CT/orthopantomogram (57%, 57%), but only in 19% of patients in the non-MRONJ group (36%, 7%) (*p* < 0.001).

### Histological and immunohistochemical findings

Tissue biopsies included necrotic bone and adjacent soft tissue without tooth or periodontal ligament structures. Histologically, inflammation was observed in nearly all osteonecrosis samples across both groups. However, a clinical suspicion of inflammation was only documented in patient files in approximately 70% of cases (Table 4). Acute inflammation appeared in 58% of samples from the MRONJ, compared to 38% in specimens from the non-MRONJ (*p* < 0.001). Chronic inflammation was observed in 27% of lesions in the non-MRONJ but in only 5% of lesions in the MRONJ (Table 4).

**Table 4:** Histological and immunohistochemical findings from tissue biopsies of patients diagnosed with jaw osteonecrosis, including antiresorptive medication-related osteonecrosis of the jaw and non-medication -related osteonecrosis of the jaw groups, along with their subgroups

Osteonecrosis type:	MRONJ: *N* = 109	MRONJ Cancer *n* = 74	MRONJ Osteoporosis *n* = 35	Non-MRONJ: *N* = 94	Non-MRONJ Osteoradio- necrosis *n* = 22	Non-MRONJ Other osteonecrosis *n* = 72	*p*-value
**Clinical inflammation**	**72%**	69%	77%	**68%**	60%	67%	*NS*
**Histopathology inflammation**	**97%**	97%	97%	**92%**	91%	92%	*NS*
***Inflammation type***:							
***Acute***	**57%**	58%	54%	**38%**	27%	42%	***< 0.001***
***Chronic***	**5%**	5%	3%	**27%**	41%	22%	***< 0.001***
*Mixed cell*	**35%**	34%	40%	**27%**	23%	28%	*NS*
***Actinomyces***:							
***Actinomyces colonies***:	**85%**	88%	80%	**51%**	50%	51%	***< 0.001***
*mild to * *moderate* ***high***	**20%** **65%**	22% 66%	17% 63%	**15%** **36%**	14% 36%	15% 36%	*NS* ***< 0.001***
***Actinomyces colonieson necrotic bone***:	**82%**	84%	77%	**47%**	46%	47%	***< 0.001***
***MMP-8***:	*N* = 103	*n* = 69	*n* = 34	*N* = 89	*n* = 20	*n* = 69	
***MMP-8: low*** *** elevated***	***31%*** ***69%***	*32%* *68%*	*29%* *71%*	***45%*** ***55%***	*45%* *55%*	*45%* *55%*	*NS* *0.048*
***MMP-8 high staining***	**61%**	61%	62%	**38%**	30%	41%	***0.002***
***MMP-8***:							
*In PMN cells*	**45%**	46%	41%	**34%**	25%	36%	*0*,*023*
***Associated with Actinomyces colonies***	**57%**	62%	47%	**34%**	35%	33%	***< 0.001***

**Osteonecrosis type: MRONJ: subgroups cancer (with bone metastases/lesions) and osteoporosis; non-MRONJ: subgroups osteoradionecrosis**,** other (causes of) osteonecrosis. Statistics**: *p-value is measured with Pearson Chi-square (2-sided) between MRONJ vs. non-MRONJ groups*

Abbreviations: *MRONJ*, medication-related osteonecrosis of the jaw; *NS*, not significant; *MMP*-8, matrix metalloproteinase-8; *PMN *cell, polymorphonuclear leucocyte.

### *Actinomyces* colonies

Histo-morphological analysis revealed *Actinomyces* colonies with typical clusters and high semi-quantitative levels in H&E and PAS staining, more frequently observed in osteonecrosis samples with MRONJ than in non-MRONJ (*p* < 0.001) (Table 4). Actinomycotic lesions were most often found lining the necrotic bone as biofilm or invading the bone, growing in cell-empty lacunae and surrounded by inflammatory cells, primarily polymorphonuclear (PMN) cells (Image 1a-d, f). Bacterial rods lining the colonies were also observed. The attachment of *Actinomyces* colonies to necrotic bone was more common in MRONJ samples than in non-MRONJ (*p* < 0.001) (Table 4). *Actinomyces* was already present in 7/8 early Stage-1 MRONJ samples. When *Actinomyces* lesions and inflammatory cell proliferation co-existed, they were visualised in close proximity throughout those samples (Image 1 c-d, f).

### Active MMP-8 and TIMP-1 immunoreactivities

Elevated levels of MMP-8 were more frequently expressed (*p* = 0.048), especially at high levels (*p* = 0.002), in lesions with MRONJ compared to non-MRONJ sites (Table 4). MMP-8 positive cells associated with *Actinomyces* lesions were observed more often in specimens with MRONJ compared to the non-MRONJ (< 0.001) (Table 4). MMP-8 immunopositivity was prominent in PMN cells but also other inflammatory cell types, most notably plasma cells and lymphocytes (Image 1f). Expression in PMN cells was more commonly seen in MRONJ than in non-MRONJ (*p* = 0.023) (Table 4).

We used the same classification to analyse TIMP-1 immunoreactivity. TIMP-1 was most often observed in stromal cells, mainly in histiocyte-like and fibroblast-like cells (Image 1e). There were no statistically significant differences in TIMP-1 expression between the studied groups (data not shown).

Logistic regression analyses were performed on the key outcomes (Actinomyces, high MMP-8 levels, and acute inflammation) for the main confounders: age, sex, current smoking, systemic corticosteroid use, and comorbidities (three or more versus two or fewer), comparing MRONJ with non-MRONJ. The analyses were performed using all samples and were repeated as a sensitivity analysis, including only the first sample from each patient to account for potential within-patient clustering (Supplementary Table 4). Logistic regression showed that the MRONJ group was independently associated with a higher prevalence of Actinomyces colonies: odds ratio = 3.469 (95% confidence interval = 1.478–8.141), *p* = 0.004. According to the logistic regression (Supplementary Table 4), the likelihood of high MMP-8 expression in osteonecrotic tissue was not explained by MRONJ: odds ratio = 2.115 (95% confidence interval = 1.004–4.458), *p* = 0.049, especially when the other separate lesion was excluded (Supplementary Table 4). Additionally, acute inflammation, which was more common in the MRONJ group than in the non-MRONJ group, could not be specifically associated with MRONJ in the logistic regression analyses: odds ratio = 1.711 (95% confidence interval = 0.811–3.612), *p* = 0.159. Current smoking was the only factor predicting acute inflammation in these analyses: odds ratio = 2.300 (95% confidence interval = 1.107—4.777), *p* = 0.026.

### Antimicrobial treatment and healing outcomes

Each patient had received many different systemic antibiotics for osteonecrosis (Table 5). The total duration of antimicrobial therapy was nearly twice as long in the MRONJ group compared to the non-MRONJ group, although individual antibiotic durations varied considerably (Table 5). All patients in the non-MRONJ group and 38/45 patients in the MRONJ group who had received cephalexin or cefuroxime had received a later antibiotic regimen effective for *Actinomyces* species.

**Table 5:** Antimicrobial therapy as cumulative duration and prescribed courses during osteonecrosis, and healing outcomes of jaw osteonecrosis in patients with tissue biopsies, categorised into medication-related osteonecrosis of the jaw and non-medication-related osteonecrosis of the jaw, including their subgroups

Osteonecrosis type:	MRONJ: *N* = 98	MRONJ Cancer *n* = 65	MRONJ Osteoporosis *n* = 33	Non-MRONJ: *N* = 93	Non-MRONJ Osteoradio-necrosis *n* = 21	Non- MRONJ Other osteonecrosis *n* = 72	*p*-value^a)^
**Cumulative duration of ab** ***(days)***: *mean ± SD*	**108** ***±*** **149**	100 ***±*** 144	123 *±* 159	**61** ***±*** **108**	94 *±* 175	51 *±* 76	*0.009*
**Antibiotics**:							
*Cephalosporins* ^*b)*^	**54**	33	21	**36**	10	26	-
*Penicillins* ^*c)*^	**74**	45	29	**40**	11	29	-
*Co-amoxiclav*	**37**	26	11	**30**	10	20	-
*Clindamycin*	**17**	11	6	**15**	3	12	-
*Broad-spectrum beta-lactam*	**8**	6	2	**2**	1	1	-
*Doxycycline*	**32**	23	9	**15**	7	8	-
*Other (FQ*,* macrolide*,* TMP-SMX etc.)*	**12**	6	6	**8**	5	3	-
*Metronidazole* ^*d)*^	**54**	36	18	**39**	10	29	-
*Fluconazole*	**3**		3	**1**	1		-
**Healing of necrosis**^**e)**^:	*N* = 103	*n* = 70	*n* = 33	*N* = 70	*n* = 17	*n* = 53	
no healing	**3%**	3%	3%	**3%**	0%	4%	
partly^f)^	**26%**	26%	27%	**11%**	18%	9%	
well after revision	**12%**	14%	6%	**9%**	0%	11%	
well without revision	**59%**	57%	64%	**77%**	82%	76%	*NS* ^g)^

**Osteonecrosis type: MRONJ-group: subgroups cancer (with bone metastases/lesions) and osteoporosis; non-MRONJ-group: subgroups osteoradionecrosis and other causes of osteonecrosis.** (a) Statistics: *p-value* is measured with *Pearson Chi-square (2-sided sig*) between MRONJ- vs. non-MRONJ-groups. **Antibiotics**,** as prescribed courses**: (b) mainly *cephalexin* po or *cefuroxime* iv, single *ceftriaxone* (c) *penicillin-G* or *-V* or *amoxicillin* (d) combined with other ab treatment. (e) see Supplementary Table 2 for “Additional separate lesions”. (f) bone contact on probing (g) statistics done together with both healed groups: well after revision and well without revision

Abbreviations: MRONJ, medication-related osteonecrosis of the jaw; *FQ*,* fluoroquinolone*; *TMP-SMX*,* trimethoprime-sulfamethoxazole; NS*,* not significant*

Among lesions in the MRONJ group, 59% healed without revision, and 12% after revision (Table 5). Of the necrotic lesions that healed and received antibiotics, 31 of 73 patients had been on systemic antibiotic therapy for up to 21 days, 22 for up to 70 days, and 20 patients for longer than 70 days (data not shown). From the non-MRONJ sites, 77% healed without revision, and 9% after revision (Table 5). In the non-MRONJ group, 22 of 60 patients with healed lesions had received a maximum of 21 days of systemic antimicrobials, 20 for up to 70 days, and 15 for over 70 days (data not shown). Partially healed lesions indicated ongoing healing, but probing revealed bone contact, necessitating follow-ups until complete healing. The average follow-up was 11 ± 12 months (median 7.0) in the MRONJ group and 8 ± 10 months (median 3.5) in the non-MRONJ group (*p* = 0.023). Of the patients, 37 disappeared from the controls, and 33 of them were from the non-MRONJ subgroup with other causes of osteonecrosis.

## Discussion

We collected clinical data from patients with osteonecrosis and available jawbone biopsies, grouping them by osteonecrosis background: medication-related osteonecrosis of the jaw (MRONJ) (*n* = 98) or non-MRONJ (*n* = 93). To our knowledge, patients with osteonecrosis have not been compared in this setting before. Interestingly, we observed several differences between these groups. Histological and immunohistochemical analyses confirmed the types of inflammation, the locations of *Actinomyces* lesions and MMP-8 activity.

Histological inflammation was observed in nearly all biopsies, with MRONJ more commonly showing acute PMN-cell type (58%) and seldom displaying chronic inflammation (5%). Conversely, non-MRONJ specimens showed a more balanced distribution of acute, chronic, and mixed cell types.

*Actinomyces* was found in almost all MRONJ samples (85%) and much more often than in non-MRONJ samples (51%). Most interestingly, we observed that *Actinomyces* colonies were localised as biofilms on the surface of necrotic bone in 82% of MRONJ samples, but significantly less often in non-MRONJ samples (47%).

Patients with MRONJ were older, more often had received corticosteroids or cytotoxic treatments, and had longer courses of antibiotics. They required surgical revision more frequently than patients with non-MRONJ lesions. MRONJ subgroups showed significant differences in co-medication use, but these variations did not affect histological or immunohistochemical findings, as results were consistent across subgroups (Table 4). Due to this heterogeneity in MRONJ patients, we conducted subgroup analyses to better understand the relationships among variables, histological results (presence and amount of *Actinomyces* colonies and inflammation type), and immunohistochemical results for MMP-8.

After adjusting for the main available confounders, the logistic regression model (Supplementary Table 4) demonstrated that MRONJ was independently associated with a higher prevalence of *Actinomyces* colonies compared to non-MRONJ. MRONJ was not independently associated with high MMP-8 immunoactivity or acute inflammation. Instead, a high prevalence of *Actinomyces* (possibly along with co-pathogens) in the MRONJ group may explain the overexpression of high levels of MMP-8 and acute inflammation in MRONJ samples, as we observed the association of inflammatory cell infiltrates with *Actinomyces* colonies and MMP-8-positive inflammatory cell proliferation with *Actinomyces* colonies in histological sections (Image 1 c-d, f). Current smoking emerged as a predictor of acute inflammation.

Our discovery of a high prevalence of *Actinomyces* colonies, primarily associated with necrotic bone as biofilm in MRONJ, aligns with previous research [8–13, 30] supporting the role of actinomycosis in the disease. However, our novel finding of a lower occurrence of *Actinomyces* colonies in non-MRONJ suggests potential differences in the pathogenesis of MRONJ and non-MRONJ. A hint of differences in the presence of *Actinomyces* in MRONJ and non-MRONJ was suggested by a small study of 11 cases [14]. Our study confirms this but also demonstrates that *Actinomyces* colonies were located on biofilm within necrotic bone and triggered an acute inflammatory response. Osteoclast-suppressive antiresorptive treatment was strongly associated with *Actinomyces* infection, as other background medications such as cytotoxic or corticosteroid treatments varied widely among patients with MRONJ.

The routine culture results from the osteonecrosis sites in our study align with previous findings of bacterial species [6–9]. *Actinomyces* was found in only 18% of the culture samples, likely due to its requirement for enriched, prolonged incubation. *Actinomyces* species have been previously detected using PCR in 37% of jaws from patients without osteonecrosis [9]. This supports the use of histopathology [12, 13, 23, 24], as in this study with Formalin-Fixed Paraffin-Embedded samples, or advanced molecular techniques [8, 9, 23, 31] or MALDI-TOF [23] from fresh samples for detecting *Actinomyces* species. Since *Actinomyces* species are nearly 100% susceptible to recommended antibiotics, sensitivity testing with culturing is unnecessary [32]. Additionally, the *Streptococcus anginosus*-group was more common in MRONJ (45%) compared to non-MRONJ (29%), possibly contributing to the pathogenesis as a biofilm-forming organism, meriting further research. *Streptococcus anginosus* has been associated with infection severity in odontogenic abscesses [33]. *Staphylococcus aureus* was, as expected, rarely detected in cultures [34].

Osteoclast-suppressive antiresorptive medication has been shown to enhance macrophage proinflammatory activity, which is thought to lead to osteocyte necroptosis and osteonecrosis, exacerbated by bacteria [17–19]. Since medication-related osteonecrosis often occurs within mucosal trauma extending to the bone, the inflammatory cascade triggered by osteoclast-suppressive therapy, *Actinomyces*, and co-bacteria [6, 17, 18] possibly delays wound and tissue healing [18, 19], as proinflammatory proteolytic enzymes such as MMP-8 can degrade collagen and basement membrane structures [28, 35, 36]. This may further promote *Actinomyces* and co-bacterial invasion, potentially increasing inflammation and osteonecrosis. Notably, MMP-8 associated with actinomycotic lesions and inflammatory cell proliferation was connected with *Actinomyces* lesions in our samples.

Since osteonecrosis occurs in both MRONJ and non-MRONJ cases, the main differences likely lie in bone immunity and homeostasis when using osteoclast-suppressive drugs. Osteoclasts function as protective osteoimmune cells [21, 22], and their suppression may facilitate opportunistic *Actinomyces* invasion on the bone surface. Moreover, inhibition of osteoclasts reduces the removal of necrotic bone and impairs bone remodelling [37], resulting in a mechanical surface that favours biofilm formation [38]. Since alveolar bone turnover is 10–20 times higher than in other bones, it becomes more susceptible to imbalance.

Active MMP-8 has been developed as a biomarker for jaw infections [27, 28]. Since inflammation was detected histologically in more cases than clinically, the MMP-8 test could assist clinicians in treatment decisions. MMP-8 levels were elevated in 70% of cases from the MRONJ group and 55% from the non-MRONJ group, and the utility and sensitivity of MMP-8 as a potential biomarker for *Actinomyces-* and co-pathogen-associated infection in MRONJ should be evaluated [27, 28].

The highest osteonecrosis risks are in males, cancer patients, those on high-dose antiresorptive medication (up to 9%) versus low dose (0,3%), corticosteroids combined with antiresorptive medication [39], and with denosumab vs. bisphosphonates [4, 39]. Despite differing risk profiles, no significant histological or immunohistochemical differences were observed between the cancer and osteoporosis groups, or between the BP and denosumab (± previous BP) groups (data not shown) in our study.

It was surprising that, despite the presence of *Actinomyces*, most patients received systemic antibiotics for less than 3 months, resulting in lesion cure. This duration is much shorter than the guidelines recommend for actinomycosis [40]. Success with the shorter systemic antibiotic regimen may be due to debridement surgery.

The limitation of our study is its retrospective nature, and patient groups could not be fully standardised. The group sizes are limited, even though we included all representative cases from the Helsinki University Hospital patient records/Biobank within the set criteria. The timing of histological sampling was based on clinical need and therefore not standardised.

In conclusion, we demonstrated that nearly all cases of osteonecrosis of the jaw showed visible inflammation. In medication-related osteonecrosis, inflammation was mainly acute, and chronic inflammation was rare. *Actinomyces* colonies were more common in MRONJ than in non-MRONJ, forming biofilm layers on the necrotic bone. We did not observe significant differences in these results across medication-related osteonecrosis subgroups, despite considerable differences in background medication. Conversely, in non-medication-related cases, chronic inflammation was more frequent, and *Actinomyces* was significantly less common. Dysregulated collagenolytic immune-cascade activity, indicated by MMP-8, was more prominent in MRONJ than in non-MRONJ. Acute inflammation and MMP-8 elevation appear to be associated with actinomycotic infection rather than directly with MRONJ, as they are found within *Actinomyces* colonies. The differences in *Actinomyces* lesions suggest that the pathophysiology of these two types of osteonecrosis may differ. Osteoclast suppression by antiresorptive medication may disrupt osteoimmunity and homeostasis, leading to opportunistic actinomycotic biofilm infections, often alongside other oral pathogens, while osteonecrosis develops through bacterial and antiresorptive medication-induced inflammatory and collagenolytic mechanisms that are both driven and amplified. Further research is necessary to determine whether *Actinomyces* is the “primus motor” in MRONJ; however, the presence of actinomycotic infection in necrotic bone with acute inflammation indicates that treatment may be warranted.

## Supplementary Information

Below is the link to the electronic supplementary material.

Supplementary Material 1

Supplementary Material 2

## Data Availability

The datasets analysed in the current study are not publicly available due to ethical and legal restrictions to protect patients’ anonymity. Patients may be potentially identifiable from individual-level data. Any other relevant data are available from the corresponding author upon reasonable request.

## Abbreviations

MRONJ: medication-related osteonecrosis of the jaw
NS: not significant
Ca: calcium
