# Supplementary material for: Actinomyces lesions and acute inflammation predominate in osteonecrosis of the jaw associated with osteoclast-suppressing therapy in contrast to non-medication-related osteonecrosis
Source: Eur J Clin Microbiol Infect Dis. 2026 Apr 6;45(7):2105–17. doi: 10.1007/s10096-026-05501-9 (PMC13328227; doi:10.1007/s10096-026-05501-9)
Supplement: Supplementary file 1 — Supplementary Material 1 [file 10096_2026_5501_MOESM1_ESM.pdf]

Supplementary material

European Journal of Clinical Microbiology & Infectious Diseases

Actinomyces lesions and acute inflammation predominate in osteonecrosis of the jaw associated with osteoclast-suppressing therapy in contrast to non-medication-related osteonecrosis

Authors

Marjo Kivelä-Rajamäki*, Hanna Välimaa, Jussi Furuholm, Caj Haglund, Timo Sorsa, Jaana Hagström, Asko Järvinen

*)

Corresponding author: Marjo Kivelä-Rajamäki Department of Infectious Diseases, Finland e-mail:marjo.kivela@helsinki.fi, marjo.kivela-rajamaki@hus.fi Address: HUS, Inflammation Centre, Division of Infectious Diseases, P.O. Box 340, 00029 HUS, Helsinki, Finland, Phone: +358-40-7351768 https://orcid.org/0009-0004-1245-3071

.

Supplementary Table 1. Immunohistochemistry protocol for antibodies.

Anti-body	Manu-facturer	Dilution[1)]	Pretreatment time (min)	Secondary Ab[2)] time (min)	DAB Chromogen[3)]	Pretreatment pH	Incubation time
**MMP-8**	Prikk et al 2002[a)]	1:300	15	20	AEC	9	ON - 4°C
**TIMP-1**	R&D AF970[b)]	1:150	10	30 GOAT	AEC Rom	9	ON - 4°C

a) non-commercial antibody, protocol according to the authors [29]. *b) R&D systems AF970, Bio-Teche, Minneapolis, MN, USA) polyclonal antibody.* 1) *Dako REAL Antibody Diluent S2022,* 2) *HRP labelled polymer secondary antibody (EnVision Flex/HRP SM802),* 3) *EnVision Flex DAB DM827, ON Counterstain (Dako Mayer´s Haematoxylin S3309.*

Abbreviations: DAB, diaminobenzidine;MMP-8, matrix metalloproteinase-8; ON, over night; TIMP-1 tissue inhibitor of metalloporeinase-1.

Supplementary Table 2. Osteonecrosis lesions and triggering events.

**Osteonecrosis type by medication: Patients N=191**	**AR: n=98**	*AR cancer: (n=65)*	*AR osteoporosis: (n=33)*	**Non-AR: n=93**	*Non-AR osteoradio-necrosis: (n=21)*	*Non-AR Other osteonecrosis: (n=72)*
**Anatomical region of the lesion: (n)**						
**Maxilla**	**10**	8	2	**0**	0	0
**Mandible**	**99**	66	33	**94**	22	72
**Additional separate lesions[a)]**	**11**	9	2	**1**	1	0
**Triggering events[b)]: (n)**						
Tooth extraction	**71**	44	27	**62**	13	49[c)]
Trauma/abrasive denture	**11**	9	2	**6**	1	5
Jaw surgery	**5**	2	3	**8**	5	3
Dental procedure	**1**	1	0	**5**	1	4
Jaw pathology	**1**	0	1	**6**	1	5
No triggering events[d)]	**20**	18	2	**7**	1	6
**- Known triggering event before AR**	**22**	11	11			
**- Known triggering event during AR**	**55**	36	19			
		mean ± SD	*mean ± SD*		*mean ±SD*	
**Timeline (months) from known triggering event to dg i.e. surgery and sampling [e)]**		20±21	47±49		19±18	*no data available*

Osteonecrosis type by medication: AR-related osteonecrosis (AR): subgroups AR cancer (with bone metastases) and AR osteoporosis; non-AR-related osteonecrosis (Non-AR): subgroups non-AR osteoradionecrosis, non-AR other (causes of) osteonecrosis. a) The additional separate lesions are from different jaw areas and occurred simultaneously or at different times. b) cases of triggering events in the anatomical area of necrosis. c) infected tooth extractions in surgery, and biopsy for osteonecrosis diagnosis taken simultaneously – no other previous triggering events except infection and poor oral hygiene. d) number of patients e).Statistics: *p-value* is measured with *Pearson Chi-square (2-sided sig)* between subgroups: cancer, osteoporosis, and osteoradionecrosis with *NS.*

Abbreviations:AR, antiresorptive medication; *NS*, not significant.

Supplementary Table 3. Laboratory results obtained before diagnostic debridement surgery.

Osteonecrosis type:	**AR**<br>**n=109**	**Non-AR**<br>**n=94**	*p=value*
Lab results: (mean ± SD)			
CRP (mg/l)	**19±30**	**32±66**	*NS*
leucocyte count (E9/l)	**12.1±21.3**	**10.5±16.7**	*NS*
Hb (g/l)	**123±16**	**136±17**	*<0.001*

Osteonecrosis type: AR-related osteonecrosis (AR): subgroups AR cancer (with bone metastases) and AR osteoporosis; non-AR-related osteonecrosis (Non-AR): subgroups non-AR osteoradionecrosis, non-AR other (causes of) osteonecrosis. Statistics: *p-value* is measured with *Pearson Chi-square (2-sided sig)* between AR- vs non-AR-groups.

Abbreviations: AR, antiresorptive medication; Lab, laboratory; CRP, C-reactive protein; Hb, haemoglobin; NS, not significant.
