# Supplementary material for: Actinomyces lesions and acute inflammation predominate in osteonecrosis of the jaw associated with osteoclast-suppressing therapy in contrast to non-medication-related osteonecrosis
Source: Eur J Clin Microbiol Infect Dis. 2026 Apr 6;45(7):2105–17. doi: 10.1007/s10096-026-05501-9 (PMC13328227; doi:10.1007/s10096-026-05501-9)
Supplement: Supplementary file 2 — Supplementary Material 2 [file 10096_2026_5501_MOESM2_ESM.pdf]

Supplementary material

European Journal of Clinical Microbiology & Infectious Diseases

Actinomyces lesions and acute inflammation predominate in osteonecrosis of the jaw associated with osteoclast-suppressing therapy in contrast to non-medication-related osteonecrosis

Authors

Marjo Kivelä-Rajamäki*, Hanna Välimaa, Jussi Furuholm, Caj Haglund, Timo Sorsa, Jaana Hagström, Asko Järvinen

*)

Corresponding author: Marjo Kivelä-Rajamäki Department of Infectious Diseases, Finland e-mail:marjo.kivela@helsinki.fi, marjo.kivela-rajamaki@hus.fi Address: HUS, Inflammation Centre, Division of Infectious Diseases, P.O. Box 340, 00029 HUS, Helsinki, Finland, Phone: +358-40-7351768 https://orcid.org/0009-0004-1245-3071

.

Supplementary Table 1. Immunohistochemistry protocol for antibodies.

Anti-body	Manu-facturer	Dilution[1)]	Pretreatment time (min)	Secondary Ab[2)] time (min)	DAB Chromogen[3)]	Pretreatment pH	Incubation time
**MMP-8**	Prikk et al 2002[a)]	1:300	15	20	AEC	9	ON - 4°C
**TIMP-1**	R&D AF970[b)]	1:150	10	30 GOAT	AEC Rom	9	ON - 4°C

a) non-commercial antibody, protocol according to the authors [29]. *b) R&D systems AF970, Bio-Teche, Minneapolis, MN, USA) polyclonal antibody.* 1) *Dako REAL Antibody Diluent S2022,* 2) *HRP labelled polymer secondary antibody (EnVision Flex/HRP SM802),* 3) *EnVision Flex DAB DM827, ON Counterstain (Dako Mayer´s Haematoxylin S3309.*

Abbreviations: DAB, diaminobenzidine; MMP-8, matrix metalloproteinase-8; ON, overnight; TIMP-1 tissue inhibitor of metalloporeinase-1.

Supplementary Table 2. Osteonecrosis lesions and triggering events.

**Osteonecrosis type by medication: Patients N=191**	**MRONJ: n=98**	*MRONJ cancer: (n=65)*	*MRONJ osteoporosis: (n=33)*	**Non-MRONJ: n=93**	*Non-MRONJ osteoradio-necrosis: (n=21)*	*Non-MRONJ Other osteonecrosis: (n=72)*
**Anatomical region of the lesion: (n)**						
**Maxilla**	**10**	8	2	**0**	0	0
**Mandible**	**99**	66	33	**94**	22	72
**Additional separate lesions[a)]**	**11**	9	2	**1**	1	0
**Triggering events[b)]: (n)**						
Tooth extraction	**71**	44	27	**62**	13	49[c)]
Trauma/abrasive denture	**11**	9	2	**6**	1	5
Jaw surgery	**5**	2	3	**8**	5	3
Dental procedure	**1**	1	0	**5**	1	4
Jaw pathology	**1**	0	1	**6**	1	5
No triggering events[d)]	**20**	18	2	**7**	1	6
**- triggering event before antiresorptive medic.**	**22**	11	11			
**- triggering event during antiresorptive medic.**	**55**	36	19			
		mean ± SD	*mean ± SD*		*mean ±SD*	*p-value*
**Time (months) from triggering to dg/sampling**		20±21	47±49		19±18	*NS*

Osteonecrosis type by medication: MRONJ: subgroups cancer (with bone metastases) and osteoporosis; non-MRONJ: subgroups osteoradionecrosis, other (causes of) osteonecrosis. a) The additional separate lesions are from different jaw areas and occurred simultaneously or at different times. b) cases of triggering events in the anatomical area of necrosis. c) infected tooth extractions in surgery, and biopsy for osteonecrosis diagnosis taken simultaneously – no other previous triggering events except infection and poor oral hygiene. d) number of patients. Statistics: *p-value* is measured with *Pearson Chi-square (2-sided sig)* between subgroups: cancer, osteoporosis, and osteoradionecrosis.
Abbreviations: MRONJ, medication-related osteonecrosis of the jaw; *NS*, not significant.

Supplementary Table 3. Laboratory results (mean±*SD)* obtained before diagnostic debridement surgery.

Osteonecrosis type:	**MRONJ n=109**	**Non-MRONJ n=94**	*p=value*
CRP (mg/l)	**19±30**	**32±66**	*NS*
leucocyte count (E9/l)	**12.1±21.3**	**10.5±16.7**	*NS*
Hb (g/l)	**123±16**	**136±17**	*<0.001*

Osteonecrosis type: MRONJ and non-MRONJ. Statistics: *p-value* is measured with *Pearson Chi-square (2-sided sig).*

Abbreviations: MRONJ, medication-related osteonecrosis of the jaw; CRP, C-reactive protein; Hb, haemoglobin.

Supplementary Table 4. Results of the multivariable binary logistic regression model predicting the likelihood of Actinomyces infection (A—B), High MMP-8 levels (C—D), and acute inflammation.

Supplementary Table 4. Results of multivariable binary logistic regression model predicting the likelihood of Actinomyces infection (A—B), High MMP-8 levels (C—D), and acute inflammation (E—F).

A) Actinomyces infection, sample-level

Variable	Coefficient	SE	OR	95% CI	p-value
Sex, male (ref. female)	0.301	0.372	1.351	0.651—2.804	0.419
Age, year	0.019	0.013	1.019	0.993—1.045	0.147
No. of comorbidites, 3 or more (ref. 2 or less)	-0.529	0.522	0.589	0.212—1.638	0.310
Current smoking, yes (ref. no)	0.340	0.405	1.406	0.636—3.108	0.400
Steroid medication, yes (ref. no)	0.440	0.431	1.553	0.667—3.615	0.307
MRONJ group, yes (ref. non-MRONJ)	1.244	0.435	3.469	1.478—8.141	**0.004**

A) Actinomyces infection, patient-level

Variable	Coefficient	SE	OR	95% CI	p-value
Sex, male (ref. female)	0.309	0.377	1.363	0.651—2.851	0.411
Age, year	0.018	0.013	1.018	0.992—1.044	0.180
No. of comorbidites, 3 or more (ref. 2 or less)	-0.578	0.531	0.561	0.198—1.588	0.276
Current smoking, yes (ref. no)	0.407	0.413	1.503	0.669—3.374	0.324
Steroid medication, yes (ref. no)	0.379	0.440	1.461	0.617—3.461	0.389
MRONJ group, yes (ref. non-MRONJ)	1.285	0.451	3.613	1.494—8.741	**0.004**

C) High MMP-8 levels, sample-level

Variable	Coefficient	SE	OR	95% CI	p-value
Sex, male (ref. female)	-0.074	0.325	0.928	0.491—1.754	0.819
Age, year	0.010	0.013	1.010	0.986—1.036	0.419
No. of comorbidites, 3 or more (ref. 2 or less)	0.317	0.472	1.373	0.544—3.464	0.502
Current smoking, yes (ref. no)	-0.437	0.361	0.646	0.318—1.310	0.226
Steroid medication, yes (ref. no)	-0.280	0.360	0.756	0.373—1.532	0.438
MRONJ group, yes (ref. non-MRONJ)	0.749	0.380	2.115	1.004—4.458	**0.049**

C) High MMP-8 levels, patient-level

Variable	Coefficient	SE	OR	95% CI	p-value
Sex, male (ref. female)	-0.207	0.334	0.813	0.423—1.563	0.534
Age, year	0.009	0.013	1.010	0.984—1.035	0.465
No. of comorbidites, 3 or more (ref. 2 or less)	0.422	0.494	1.525	0.579—4.020	0.393
Current smoking, yes (ref. no)	-0.484	0.371	0.617	0.298—1.275	0.192
Steroid medication, yes (ref. no)	-0.222	0.374	0.801	0.385—1.668	0.553
MRONJ group, yes (ref. non-MRONJ)	0.716	0.394	2.047	0.945—4.432	0.069

E) Acute inflammation, sample-level

Variable	Coefficient	SE	OR	95% CI	p-value
Sex, male (ref. female)	0.186	0.327	1.204	0.635—2.284	0.569
Age, year	0.025	0.013	1.025	1.000—1.051	**0.048**
No. of comorbidites, 3 or more (ref. 2 or less)	0.173	0.457	1.188	0.486—2.908	0.705
Current smoking, yes (ref. no)	0.833	0.373	2.300	1.107—4.777	**0.026**
Steroid medication, yes (ref. no)	-0.036	0.355	0.965	0.481—1.936	0.920
MRONJ group, yes (ref. non-MRONJ)	0.537	0.381	1.711	0.811—3.612	0.159

E) Acute inflammation, patient-level

Variable	Coefficient	SE	OR	95% CI	p-value
Sex, male (ref. female)	0.145	0.332	1.157	0.604—2.215	0.661
Age, year	0.021	0.013	1.021	0.996—1.047	0.096
No. of comorbidites, 3 or more (ref. 2 or less)	0.094	0.469	1.099	0.439—2.754	0.840
Current smoking, yes (ref. no)	0.840	0.378	2.317	1.103—4.864	**0.026**
Steroid medication, yes (ref. no)	-0.101	0.366	0.904	0.441—1.854	0.783
MRONJ group, yes (ref. non-MRONJ)	0.619	0.393	1.857	0.860—4.013	0.115

SE = standard error
OR = odds ratio
CI = confidence interval
